# Supplementary material for: The ANTsX ecosystem for mapping the mouse brain
Source: Nat Commun. 2025 Nov 22;16:11548. doi: 10.1038/s41467-025-66741-5 (PMC12748665; doi:10.1038/s41467-025-66741-5)
Supplement: Supplementary file 1 — Reporting Summary [file 41467_2025_66741_MOESM1_ESM.pdf]

nature portfolio

Corresponding author(s): Nicholas J. Tustison
James C. Gee

Last updated by author(s): Oct 23, 2025

Reporting Summary

Nature Portfolio wishes to improve the reproducibility of the work that we publish. This form provides structure for consistency and transparency in reporting. For further information on Nature Portfolio policies, see our Editorial Policies and the Editorial Policy Checklist.

Statistics

For all statistical analyses, confirm that the following items are present in the figure legend, table legend, main text, or Methods section.

n/a	Confirmed	
☐	☒	The exact sample size (*n*) for each experimental group/condition, given as a discrete number and unit of measurement
☐	☒	A statement on whether measurements were taken from distinct samples or whether the same sample was measured repeatedly
☒	☐	The statistical test(s) used AND whether they are one- or two-sided<br>*Only common tests should be described solely by name; describe more complex techniques in the Methods section.*
☒	☐	A description of all covariates tested
☒	☐	A description of any assumptions or corrections, such as tests of normality and adjustment for multiple comparisons
☐	☒	A full description of the statistical parameters including central tendency (e.g. means) or other basic estimates (e.g. regression coefficient) AND variation (e.g. standard deviation) or associated estimates of uncertainty (e.g. confidence intervals)
☒	☐	For null hypothesis testing, the test statistic (e.g. F, t, r) with confidence intervals, effect sizes, degrees of freedom and P value noted<br>*Give P values as exact values whenever suitable.*
☒	☐	For Bayesian analysis, information on the choice of priors and Markov chain Monte Carlo settings
☒	☐	For hierarchical and complex designs, identification of the appropriate level for tests and full reporting of outcomes
☒	☐	Estimates of effect sizes (e.g. Cohen's *d*, Pearson's *r*), indicating how they were calculated

Our web collection on statistics for biologists contains articles on many of the points above.

Software and code

Policy information about availability of computer code

Data collection

No new data were collected for this study. All datasets were previously published and acquired from public repositories (e.g., Allen Institute, Brain Image Library, OpenNeuro, FRDR, Mendeley Data). Raw data were acquired using previously described imaging instruments (e.g., MRI scanners, light sheet microscopes) as detailed in the source publications. The current work focuses on processing and analysis using existing datasets.

Data analysis

All data analysis was performed using open-source tools from the ANTsX software ecosystem:

* ANTs (Advanced Normalization Tools): https://github.com/ANTsX/ANTs, version 2.4.4
* ANTsPy/ANTsPyNet: https://github.com/ANTsX/ANTsPy, https://github.com/ANTsX/ANTsPyNet, versions 0.2.6 / 0.3.7
* ANTsR/ANTsRNet: https://github.com/ANTsX/ANTsR, https://github.com/ANTsX/ANTsRNet, versions 0.5.2 / 0.7.3

Additional code (custom Python and R scripts) used in this manuscript is available at:

* https://github.com/ntustison/ANTsXMouseBrainMapping
* https://github.com/dontminchenit/CCFAlignmentToolkit

Data visualization was performed using Matplotlib (v3.8), ggplot2 (v3.4.0), and ITK-SNAP (v3.8.0) for interactive anatomical inspection.

For manuscripts utilizing custom algorithms or software that are central to the research but not yet described in published literature, software must be made available to editors and reviewers. We strongly encourage code deposition in a community repository (e.g. GitHub). See the Nature Portfolio guidelines for submitting code & software for further information.

Data

Policy information about availability of data

All manuscripts must include a data availability statement. This statement should provide the following information, where applicable:

- Accession codes, unique identifiers, or web links for publicly available datasets
- A description of any restrictions on data availability
- For clinical datasets or third party data, please ensure that the statement adheres to our policy

The following datasets were used in this study and are publicly available:

* **Allen Common Coordinate Framework (AllenCCFv3)**: Available from the Allen Institute for Brain Science at https://atlas.brain-map.org/atlas.
* **Developmental Common Coordinate Framework (DevCCF)** MRI and LSFM datasets: Publicly available via the Kim Lab https://kimlab.io/home/projects/DevCCF/index.html.
* **MERFISH spatial transcriptomics data**: Previously published [@Yao:2023aa] https://portal.brain-map.org.
* **Developmental datasets for brain extraction and segmentation**:
  * High-resolution MRI data of brain C57BL/6 and BTBR mice in three different anatomical views: https://data.mendeley.com/datasets/dz9x23fttt/1.
  * CAMRI Mouse Brain Data: https://openneuro.org/datasets/ds002868/versions/1.0.1
* **Evaluation dataset for brain extraction and segmentation**: A longitudinal microstructural MRI dataset in healthy C57Bl/6 mice at 9.4 Tesla https://www.frdr-dfdr.ca/repo/dataset/9ea832ad-7f36-4e37-b7ac-47167c0001c1.
* **ANTsXNet-pretrained templates and models**:
 Available through ANTsPy at [https://github.com/ANTsX/ANTsPyNet](https://github.com/ANTsX/ANTsXNet).

Research involving human participants, their data, or biological material

Policy information about studies with human participants or human data. See also policy information about sex, gender (identity/presentation), and sexual orientation and race, ethnicity and racism.

Reporting on sex and gender	Not applicable. This study does not involve human participants.
Reporting on race, ethnicity, or other socially relevant groupings	Not applicable. This study does not involve human participants.
Population characteristics	Not applicable. This study does not involve human participants.
Recruitment	Not applicable. This study does not involve human participants.
Ethics oversight	Not applicable. No human data were collected or analyzed.

Note that full information on the approval of the study protocol must also be provided in the manuscript.

Field-specific reporting

Please select the one below that is the best fit for your research. If you are not sure, read the appropriate sections before making your selection.

☒ Life sciences ☐ Behavioural & social sciences ☐ Ecological, evolutionary & environmental sciences

For a reference copy of the document with all sections, see nature.com/documents/nr-reporting-summary-flat.pdf

Life sciences study design

All studies must disclose on these points even when the disclosure is negative.

Sample size	Sample sizes were determined by the full availability of open, curated datasets used in this work, including the DevCCF, MERFISH, fMOST, and MRI parcellation evaluation cohorts. Each dataset encompasses the complete set of specimens released for that modality, ensuring comprehensive coverage rather than selective sampling. Because the study's goal was to develop and validate image registration and segmentation workflows rather than test biological hypotheses, formal power calculations were not applicable. The number and diversity of images across these datasets are sufficient to demonstrate pipeline robustness and reproducibility.
Data exclusions	No data were excluded from analysis. All available subjects from each dataset were included unless relevant modalities were missing (e.g., absence of structural imaging needed for registration).
Replication	All pipelines were implemented using version-controlled open-source code, and full processing scripts, model weights, and figures are

Replication	available at https://github.com/ntustison/ANTsXMouseBrainMapping . Results are reproducible on public datasets using the provided commands. Key pipelines (e.g., DevCCF velocity model, deep learning parcellation) were evaluated on independent held-out datasets, demonstrating generalizability and robustness.
Randomization	Not applicable. No experimental groups or treatment conditions were used. This study analyzes publicly available imaging data using uniform processing pipelines.
Blinding	Not applicable. No group allocation or subjective manual scoring was involved. All analyses were conducted using automated methods.

Reporting for specific materials, systems and methods

We require information from authors about some types of materials, experimental systems and methods used in many studies. Here, indicate whether each material, system or method listed is relevant to your study. If you are not sure if a list item applies to your research, read the appropriate section before selecting a response.

Materials & experimental systems

n/a	Involved in the study
☒	☐ Antibodies
☒	☐ Eukaryotic cell lines
☒	☐ Palaeontology and archaeology
☐	☒ Animals and other organisms
☒	☐ Clinical data
☐	☒ Dual use research of concern
☒	☐ Plants

Methods

n/a	Involved in the study
☒	☐ ChIP-seq
☒	☐ Flow cytometry
☐	☒ MRI-based neuroimaging

Animals and other research organisms

Policy information about studies involving animals; ARRIVE guidelines recommended for reporting animal research, and Sex and Gender in Research

Laboratory animals	This study uses publicly available mouse brain datasets from C57BL/6 and BTBR strains. Developmental imaging spans from embryonic day 11.5 (E11.5) to postnatal day 56 (P56). Sex was not a factor in the analyses and was not consistently specified across datasets.
Wild animals	Not applicable. No wild animals were used.
Reporting on sex	Sex was not analyzed as a variable in this study. Some datasets included mixed-sex samples, but sex-specific effects were not a focus of this work.
Field-collected samples	Not applicable. No field-collected samples were used.
Ethics oversight	No new animal experiments were conducted. All data were acquired from previously published studies that received institutional animal care and use approval (e.g., Allen Institute for Brain Science, BICCN, CAMRI, and collaborating institutions).

Note that full information on the approval of the study protocol must also be provided in the manuscript.

Plants

Seed stocks	Not applicable.
Novel plant genotypes	Not applicable.
Authentication	Not applicable.

Magnetic resonance imaging

Experimental design

Design type	Not applicable. fMRI is not used in this study.
Design specifications	Not applicable. fMRI is not used in this study.
Behavioral performance measures	Not applicable. fMRI is not used in this study.

Acquisition

Imaging type(s)	Structural
Field strength	DevCCF: 7T (mouse brain developmental MRI)<br>Evaluation dataset: 9.4T (longitudinal C57BL/6 dataset, FRDR)<br>CAMRI data: 9.4 T
Sequence & imaging parameters	Sequence parameters vary by dataset:<br><br>DevCCF: Primarily T2-weighted structural imaging (see https://kimlab.io/home/projects/DevCCF/index.html for details)<br><br>CAMRI and Mendeley datasets: T1- and T2-weighted imaging; specific TR/TE values are provided in their respective repositories<br><br>Evaluation dataset: T2-weighted microstructural imaging (see FRDR record for acquisition details)<br><br>This study used these data in processed form; original acquisition parameters are detailed in the cited source publications.
Area of acquisition	All MRI data included full-brain coverage of the mouse brain.

Diffusion MRI ☐ Used ☒ Not used

Preprocessing

Preprocessing software	Preprocessing was performed using tools from the ANTsX ecosystem:<br>* ANTs (v2.4.4)<br>*ANTsR/ANTsRNet (v0.5.2 / 0.7.3)<br>*ANTsPy/ANTsPyNet (v0.2.6 / 0.3.7)<br><br>Code and pipelines are available at:<br>https://github.com/ntustison/ANTsXMouseBrainMapping
Normalization	Structural images were spatially normalized using ANTs-based deformable image registration. For developmental data, a continuous velocity flow model aligned each sample to the DevCCF template at the appropriate developmental timepoint. For evaluation, ANTs multi-component registration was used to map images to the AllenCCFv3 space.
Normalization template	*DevCCF developmental templates (E11.5 to P56) from https://kimlab.io/home/projects/DevCCF/index.html<br>*AllenCCFv3 for adult atlas alignment, available at https://atlas.brain-map.org/atlas
Noise and artifact removal	Standard ANTsX preprocessing tools were applied to correct for intensity inhomogeneity (N4 bias field correction) and reduce noise (non-local means denoising or deep learning–based denoising, depending on dataset). For multi-slice or LSFM-derived data (e.g., MERFISH, fMOST), additional custom alignment steps were applied to correct for slice shifts or distortions. Motion correction was not required, as all MRI datasets were ex vivo or under tightly controlled imaging conditions.
Volume censoring	Not applicable. No fMRI or time-series data were used in this study. All MRI datasets were structural and acquired under ex vivo or low-motion conditions. Volume censoring was not required.

Statistical modeling & inference

Model type and settings	No statistical models were used for inference. The study focused on computational image registration, velocity field modeling, and deep learning–based parcellation using ANTsX tools. Evaluation was based on descriptive overlap metrics (e.g., Dice coefficient) between anatomical label sets.
Effect(s) tested	No statistical effects were tested. The study evaluated the performance of image processing pipelines across developmental stages and imaging modalities. Comparative metrics (e.g., Dice overlap between pairwise SyN registration and velocity flow interpolation) were used to assess alignment quality.

Specify type of analysis: ☐ Whole brain ☐ ROI-based ☒ Both

Anatomical location(s)	Both. Whole-brain processing was performed for image registration, segmentation, and parcellation tasks. ROI-based evaluation was conducted using anatomical regions defined by the Allen Common Coordinate Framework version 3 (AllenCCFv3) and the Developmental Common Coordinate Framework (DevCCF), including cerebral cortex, hippocampus, cerebellum, olfactory bulb, brainstem, and subcortical nuclei.
Statistic type for inference<br>(See Eklund et al. 2016)	Not applicable. No inference maps or statistical tests were performed. Evaluation relied on descriptive metrics such as the Dice similarity coefficient for anatomical overlap.
Correction	No correction for multiple comparisons was applied. The study did not perform statistical hypothesis testing. Evaluation was based on descriptive overlap metrics (e.g., Dice coefficient) and qualitative anatomical assessments.

Models & analysis

n/a	Involved in the study
☒	☐ Functional and/or effective connectivity
☒	☐ Graph analysis
☒	☐ Multivariate modeling or predictive analysis
